# SERS Investigation of Cancer Cells Treated with PDT: Quantification of Cell Survival and Follow-up

**DOI:** 10.1038/s41598-017-07469-1

**Published:** 2017-08-03

**Authors:** A. B. Veloso, J. P. F. Longo, L. A. Muehlmann, B. F. Tollstadius, P. E. N. Souza, R. B. Azevedo, P. C. Morais, S. W. da Silva

**Affiliations:** 1 Institute of Physics, University of Brasília, Brasília, DF 70910-900 Brazil; 2 Institute of Biological Sciences, University of Brasília, DF 70910-900 Brasília, Brazil; 3 School of Chemistry and Chemical Engineering, Anhui University, Hefei, 230601 China

## Abstract

In this study Surface Enhanced Raman Spectroscopy (SERS) data recorded from mouse mammary glands cancer cells (4T1 cell line) was used to assess information regarding differences between control, death and viable cells after Photodynamic Therapy (PDT) treatment. The treatment used nanoemulsions (NE/PS) loaded with different chloroaluminumphthalocyanine (ClAlP) photosensitizer (PS) contents (5 and 10 µmol × L^−1^) and illumination (660 nm wavelength) at 10 J × cm^−2^ (10 minutes). The SERS data revealed significant molecular alterations in proteins and lipids due to the PDT treatment. Principal Component Analysis (PCA) was applied to analyze the data recorded. Three-dimensional and well reproductive PCA scatter plots were obtained, revealing that two clusters of dead cells were well separated from one another and from control cluster. Overlap between two clusters of viable cells was observed, though well separated from control cluster. Moreover, the data analysis also pointed out necrosis as the main cell death mechanism induced by the PDT, in agreement with the literature. Finally, Raman modes peaking at 608 cm^−1^ (proteins) and 1231 cm^−1^ (lipids) can be selected for follow up of survival rate of neoplastic cells after PDT. We envisage that this finding is key to contribute to a quick development of quantitative infrared thermography imaging.

## Introduction

Worldwide, breast cancer is the most common cancer among women. In 2012, 1.67 million new cases were recorded, representing 25% of women diagnosed cancer^1^. Treatments most commonly adopted for breast cancer are surgery, radiotherapy, chemotherapy, or a combination of these therapies. Photodynamic therapy (PDT) emerges as a new and less aggressive approach that can replace or support conventional treatments^2–6^. Currently, the PDT is used for superficial cancer treatments such as mouth and skin, including breast cancer in Stage III while spread to chest wall or breast skin^3, 6^. However, emerging nanostructured materials, new photosensitizers or even deeply penetrating lasers can successfully extend the use of the PDT to breast cancer^7^. *In situ* production of oxidative species by a photosensitizer (PS) while optically excited is the basis of the PDT, as the PS is capable of convert light into chemical energy. In aerobic cells, the PS converts triplet O_2_ into singlet O_2_, the latter being a strong oxidant agent (reactive oxygen species) and thus able to cause enormous oxidative stress while generated in high amounts^7^. Such oxidative stress can be high enough to promote cellular death or generate significant responses to it. Living cells can react in different ways under stressing stimuli. It may activate survival mechanisms or undergo death when survival mechanisms fail to restore cell homeostasis. Initially, the cells’ response to stressing stimuli is focused on defense and/or recovery from damages^8^. However, if stress is too severe or prolonged cells will not be able to defend themselves, leading to activation of death mechanisms. How do cells respond to stressing situation depends on the cell type and level of stress. In case of cell death, the activation of a death pathway depends on cell’s ability to deal with exposed conditions, while surviving depends on cell’s ability to create an adequate response to stimulus^8^. Reactive oxygen species (ROS) are among the most potent threats faced by living cells, as they can damage major classes of biological macromolecules, including proteins, lipids, nucleic acids, and carbohydrates^8^. Therefore, when antioxidant cells defenses are overloaded, ROS can induce cell death, and the imbalance between oxidants and antioxidants determines not only cell fate, but also the death mechanism. Several studies show that ROS, such as superoxide anion (*O*_*2*_^−^), hydrogen peroxide (H_2_O_2_) and nitric oxide (NO) are capable of triggering cell death by apoptosis. However, ROS can also interfere with the apoptotic death program causing cells death by necrosis^8^. The anticancer mechanisms related to the PDT include direct induction of cancer cell death^9–11^, vascular shutdown with subsequent tumor ischemia^12^, and activation/stimulation of immune responses against tumor antigens^3, 7^. However, it is known that the PDT can also induce generation of survivor clones with more aggressive behavior, increasing the progression and aggressiveness of the tumor cells who survived the treatment^3^. In this regard it is of high interest to develop a robust protocol that quantitatively assess the fraction of generated resistant clones in the short follow up term of the PDT treatment.

Raman spectroscopy has demonstrated to be a powerful tool for identification and quantification of chemical species present in a sample, since the set of vibrational modes of a particular molecule or crystal is uniquely related to its chemical identity^13, 14^. Raman spectroscopy is extremely useful for a wide range of applications and it has been used in qualitative and quantitative analytical studies in the fields of chemistry, biology, geology, pharmacology, solid state physics among others^15–17^. In biology, Raman spectroscopy has been widely used for identification and quantification of biomolecules^18–20^. For example, the technique was successfully used to distinguish between cancerous and non-cancerous cells and tissues^21, 22^. However, conventional Raman spectroscopy presents limitations in the investigation of biosystems as many biomolecules have a low cross-section and intense fluorescence effects. Therefore, in recent years more advanced experimental approaches were introduced to overcome those limitations and, in this context, Surface Enhanced Raman Spectroscopy (SERS) emerged as a powerful and innovative analytical tool for the study of biological systems^23, 24^. The SERS effect may enhance the Raman signal by a factor that can exceed 10^6^, occurring when the target molecule is absorbed or placed nearby nanostructured metal surfaces^25, 26^. Therefore, the SERS technique can be very much useful as a tool to investigate and discriminate cancer cells subjected to different therapies, in particular the PDT^27–29^. Despite the huge sensitivity provided by the SERS, spectral differences between cancerous and non-cancerous cells are very subtle, often hard to notice by direct analysis of the spectra, thus requiring a more robust approach that can be provided by statistical analysis. In this context, Raman spectroscopy combined with multivariate analysis, such as Principal Component Analysis (PCA), has been successfully used to distinguish different types of cancer cells and even different stages of cancer^30–32^.

**Figure 1:**
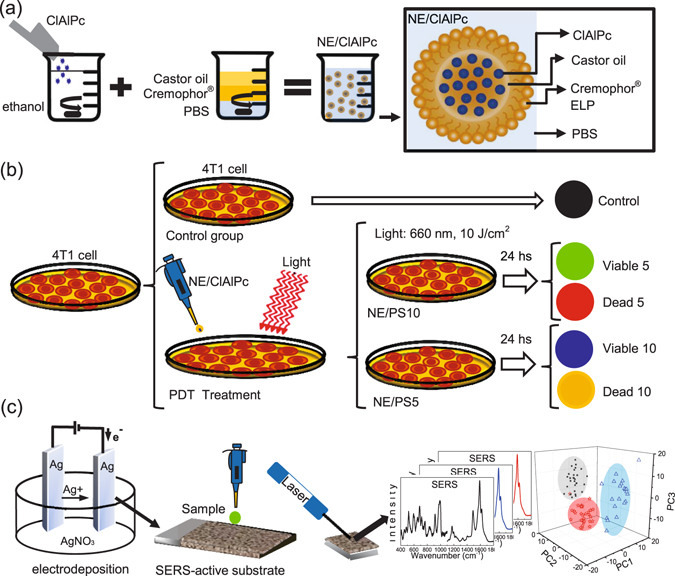
Schematic diagram of the nanoemulsion complex preparation (drug carrier system) (**a**), the PDT protocol used in the *in vitro* assay (**b**), and the SERS probe approach (**c**).

In the present study, we report on results of SERS and PCA combined in the investigation of mouse adenocarcinoma breast cancer cells (4T1 line) treated with the PDT. A nanoemulsion, comprising monodisperse nanodroplets of castor oil containing the PS chloroaluminumphthalocyanine (NE/ClAlPc) and stabilized by Cremophor ELP^®^^33^, was fabricated and used in the PDT protocol to treat breast cancer cells (4T1 line) cultured *in vitro*. For the PDT treatment (see Fig. 1a) the nanoemulsion was prepared with two different PS concentrations, namely 5 and 10 µmol × L^−1^ of ClAlPc and labeled NE/PS5 and NE/PS10 complexes, respectively. After the PDT treatment the cells were separated into two groups (see Fig. 1b: (*i*) viable cells, which were kept attached to the bottom flask and (*ii*) dead cells, considered here as the cells in suspension within the culture medium. Untreated cells were used as control group and the five cell groups were frozen in liquid Nitrogen for further SERS measurements). Electrodeposited Silver films were used for the SERS evaluation whereas the multivariate analysis was performed by PCA (see Fig. 1c).

## Results

**Figure 2:**
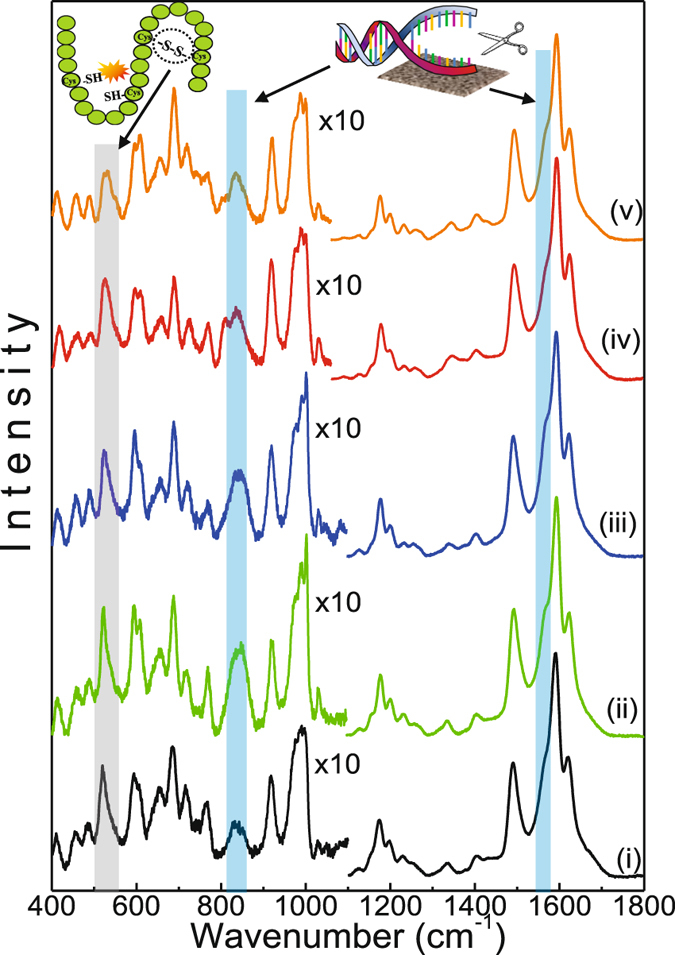
Average SERS spectra of 4T1 breast cancer cells for control group and cells treated with the NE/PS5 and NE/PS10 complexes. Each spectrum represents an average of 30 spectra recorded from cellular samples. In the low energy region (400–1100 cm^−1^) the band intensities were multiplied by 10. The inserted pictures emphasize the vibrational modes related to the protein disulfide bonds (-S-S-) at ~525 cm^−1^ and DNA fragmentation responsible for the SERS intensity variation associated with phosphodiester bonds (~813 cm^−1^) and guanine/adenine bases (~1565 cm^−1^).

Figure 2 shows the average SERS spectra recorded from the 4T1 cells after the PDT treatment using both NE/ClAlPc formulations (NE/PS5 and NE/PS10). The average SERS spectrum of the control group is also presented in Fig. 2 (lower spectrum). Thirty spectra were recorded from samples collected from each cell group and were normalized to their integrated area in the range of 400–1800 cm^−1^. All spectra in Fig. 2 show signatures of well-defined patterns, displaying cellular fingerprints consisting of proteins, lipids, and nucleic acid complexes^34^.

Overall, the SERS spectra shown in Fig. 2 are very much similar, with slight variations in intensity and peak position. In general, for vibrational modes around 765 cm^−1^ (Trp), 1122 cm^−1^ (CN peptide), 1173 cm^−1^ (Tyr), 1250 cm^−1^ (amide III), 1337 (CH proteins) and 1623 cm^−1^ (Trp/Tyr), the SERS spectra of the cells treated with the PDT protocol (viable and dead) show Raman shifts to higher energy when compared to the control group. The assignments of the vibrational modes were based on a search in the literature^34–37^ and are summarized in Table 1. It is worth mentioning that we found higher Raman shifts for vibrational modes associated with the dead cells groups and lower Raman shifts associated with the viable cells groups. In addition, most of the bands revealing Raman shifts to higher energy are associated with protein vibrational modes.

**Table 1:** SERS bands observed in the spectra recorded from the 4T1 breast cancer cells and the corresponding tentative assignments.

Raman peak [cm^−1^]	Assignments			
	Protein	Lipid	DNA	Other
410		PtdIns^&^		
456	ring torsion, Phe^&^			
485			DNA^&^	
520	(-S-S-)^&^			
533	C=C^&^			
549	(-S-S-)^&^			
586				OH^&^
593		PtdIns^&^		
608	C-C, twist Phe^&^			
638	C-C, twist Tyr^&^			
655			T, G*	CH_2_, out-plane bend^+^
685				ring def
718		C-N*^§^ Phospholipid		
765	Trp*			
813			str (O-P-O)*	
828	ring breath, Tyr^§^		str ass (O-P-O)*	
846	ring breath, Tyr^§^			
973	C-C, str-sheet*	CH bend*		
1003	Phe*^§^			
1030	Phe*^§^			
1051		(C–C) str* ^§^		C-O, C-C*
1077			PO_2_^−§^	
1122	C-N, str Peptide^§^			
1153	C-C/C-N, str* ^§^			Glycogen^+^
1173	C-H, bend Tyr*			
1199	C-C_6_H_4_, Phe, Trp^+^			
1230		CH bend^§^		
1253	Amide III*^§^		T, A*	
1325	CH, def*		G*	
1337	CH, def^+^			
1375			A, G^+^	
1404	CH_3_, colog^+^			Triglyceride^+^
1434		CH def*	G, A, CH, def*	
1488	CH, def ^§^		DNA^+^	
1500	CH, in plane^&^			
1565			G, A^+^	
1591	C-N, NH_2_^+^			
1623	C=C, Tyr, Trp^+^			
1661	Amide I*	str (C=C)*		

Ref. 33*; 34 ^+^; 35 ^§^; 36 ^&^ (Phe: Phenylalanine; Tyr: Tyrosine, Trp: Tryptophan; PtdIns: Phosphatidylinositid).

**Figure 3:**
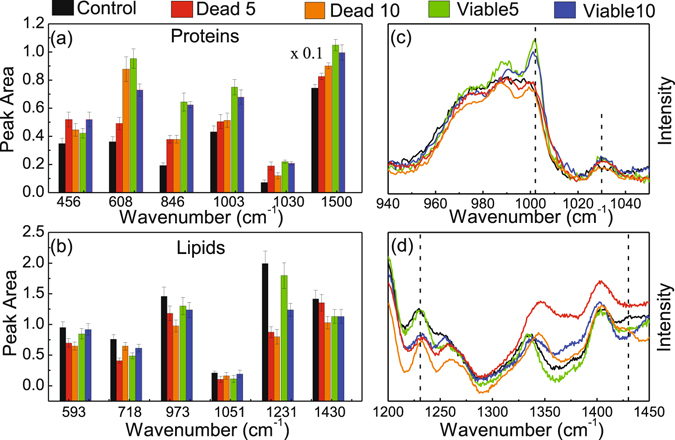
Comparison between the values of average intensities and standard deviations of SERS peaks related to proteins (**a**) and lipids (**b**). Spectral details are shown in (**c** and **d**) for proteins and lipids, respectively.

Important to note in the SERS spectra of the dead cells a new Raman band peaking at 813 cm^−1^ in addition to intensity reduction of the 1565 cm^−1^ Raman band associated with DNA vibration (phosphodiester bonds O-P-O in the backbone of DNA) and adenine/guanine bases, respectively (see light-blue strip lines in Fig. 2)^38^. Interestingly, the new 813 cm^−1^ Raman band is more intense in the spectra of the cell group treated with the NE/PS5 complex compared to the NE/PS10 complex. Moreover, in comparison with the control group the SERS spectra of all PDT treated groups showed increased intensity of the Raman features related to proteins, namely at 456 cm^−1^ (Phe), 608 cm^−1^ (Phe), 846 cm^−1^ (Tyr), 1003 cm^−1^ (Phe), 1030 cm^−1^ (Phe), and 1500 cm^−1^ (Phy). Actually, we found this intensity increase is even higher for the viable cells groups. However, no intensity increase pattern was observed for the Raman bands peaking at 520 and 549 cm^−1^, both associated with protein disulfide bonds (-S-S-) vibrations. In this case (520 and 549 cm^−1^ features), the intensity of the Raman bands remained nearly constant for both viable cells groups and revealed a strong intensity reduction for both dead cells groups (~40% and 60% for dead cells groups treated with the NE/PS5 and NE/PS10 complexes, respectively). Finally, intensity reduction of vibrational modes associated with lipids and peaking at 593 cm^−1^ (PtdIns), 718 cm^−1^ (CN fat), 973 cm^−1^ (CH lipids), 1051 cm^−1^ (CC lipids), 1231 cm^−1^ (CH lipids) and 1434 cm^−1^ (CH lipids) were also observed. For comparison, Fig. 3(a,b) collect the relative Raman peak intensity data of the bands discussed above and related to proteins and lipids, respectively. Likewise, Fig. 3(c,d) show spectral details related to selected vibrational modes of proteins and lipids, respectively.

While comparing with the control group, the SERS spectral variations of viable cells groups (treated with the NE/PS5 and NE/PS10 complexes) are higher for proteins and lower for lipids (see Fig. 3), indicating some kind of cellular stress after contact between lipid nanodroplets and cell membrane. With respect to the control group, the average total integrated areas of vibrational modes related to proteins increased 42% for the dead cells groups (35% and 50% for treatments using the NE/PS5 and NE/PS10 complexes, respectively) and 80% for the viable cells groups (85% and 75% for the NE/PS5 and NE/PS10 complexes, respectively). On the other hand, the reduction of the total integrated areas related to vibrational modes of lipids decreased on average 35% (32% and 37% for treatments using the NE/PS5 and NE/PS10 complexes, respectively) and 20% (18% and 22% for the NE/PS5 and NE/PS10 complexes, respectively) for the dead and viable cells groups, respectively. The SERS spectral variation related to vibrational modes of nucleic acids were also lower for the viable cells groups compared with the dead cells groups. The key difference we found is the Raman peak at 813 cm^−1^ observed only in the SERS spectra of the dead cells groups and assigned to DNA vibration.

**Figure 4:**
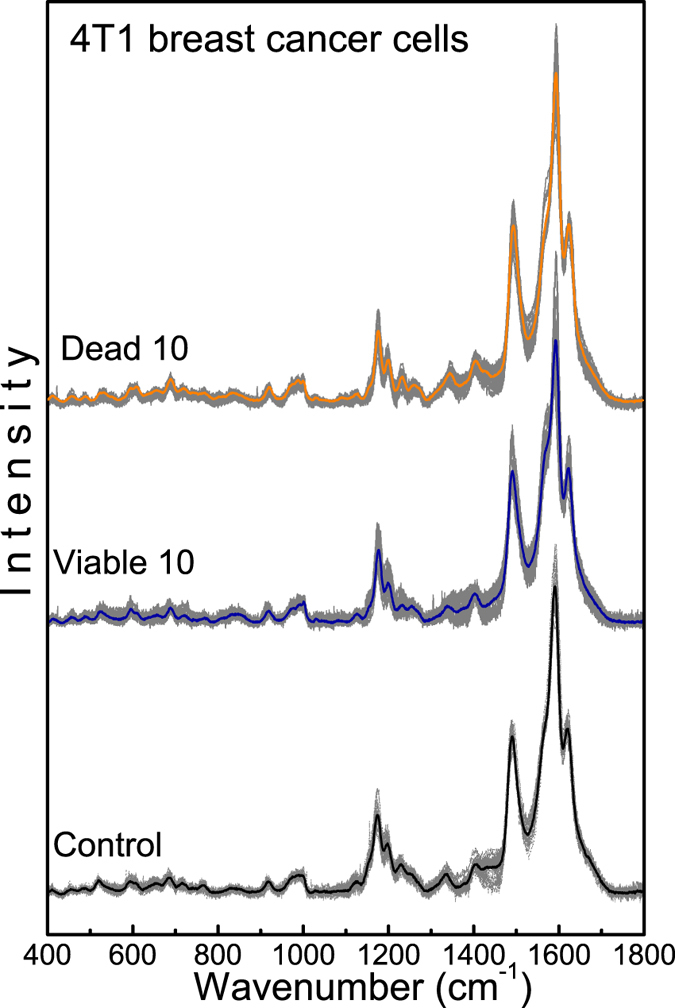
SERS spectra of 4T1 breast cancer cells groups: control and viable and dead cells PDT treated using the NE/PS10 complex. Solid lines are spectral averages.

To determine whether SERS can be used or not to distinguish from the control group the cells treated (viable and dead cells groups) with the PDT protocol (using the NE/PS5 and NE/PS10 complexes), Principal Component Analysis (PCA) was employed to analyze the recorded spectra. The covariance matrix was constructed from 150 SERS spectra (all spectral points - 30 spectra for each cell group) to determine the variance between different cells groups. Thus, as in Fig. 2, the principal components (PCs) in the range of 400–1800 cm^−1^ were extracted after baseline correction and normalization with respect to the spectral areas. A typical example of the variance spectrum is shown in Fig. 4 for the control group and the PDT treated cells groups (viable and dead), where solid lines represent spectral averages.

**Figure 5:**
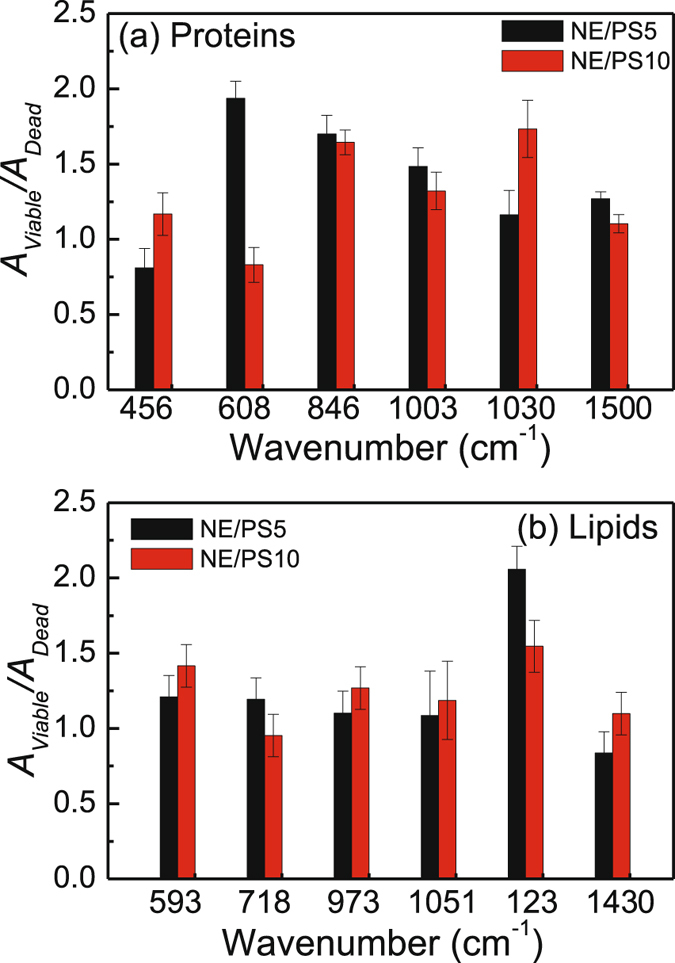
Raman peak area ratio (viable/dead cells) related to (**a**) proteins and (**b**) lipids. Data were collected for cells treated using both complexes (NE/PS5 and NE/PS10).

Figure 5(a,b) collect the ratio of Raman peak areas (SERS) related to viable/dead cells (*A*_*viable*_/*A*_*dead*_) for the vibrational modes explored in Fig. 3 and related to proteins and lipids, respectively. Note that the data in Fig. 5(a,b) were organized for cells treated using both complexes NE/PS5 and NE/PS10.

**Figure 6:**
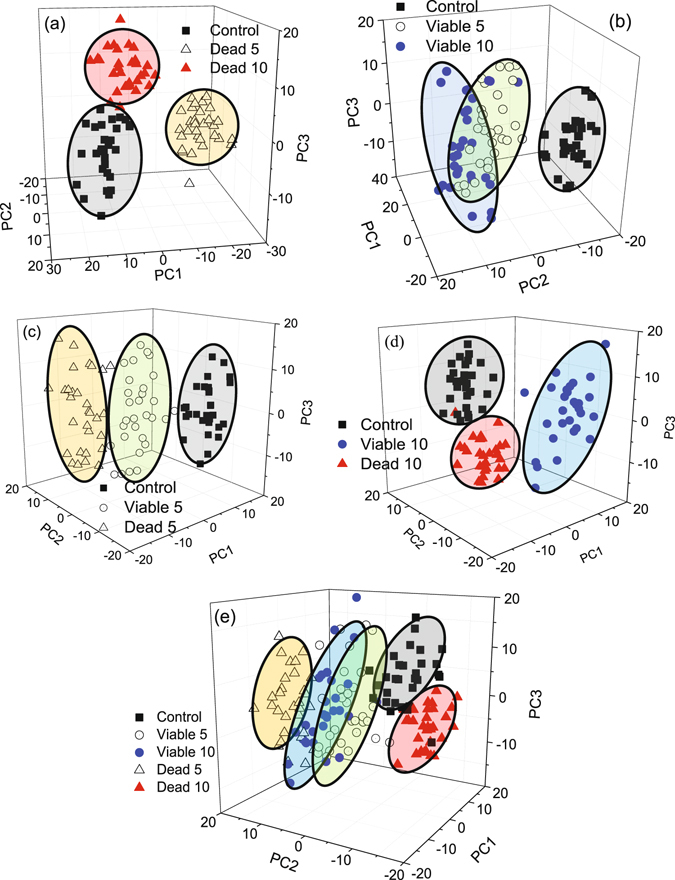
PCA scatter plots of the 4T1 breast cancer cells groups: control and dead (**a**), control and viable (**b**), control and treated with NE/PS5 (**c**), and control and treated with NE/PS10 (**d**). A comparison among all five cells groups is shown in (**e**).

Figure 6 shows the dispersion points using the first three PCs, which represent variances exceeding 41%. Notice the formation of well-defined sets in the PC space, corresponding to control cells group (black squares), dead cells groups (open and solid triangles for the NE/PS5 and NE/PS10 complexes, respectively), and viable cells groups (open and solid circles for the NE/PS5 and NE/PS10 complexes, respectively). In comparison with the control cells group Fig. 6(a,b) show the first three PCs, representing 45% (dead cells groups) and 43% (viable cells groups) of data variance, respectively. Worth mentioning that while the clusters of each dead cells groups are well separated there is an overlap between the two groups of viable cells treated with different PS doses (NE/PS5 and NE/PS10 complexes). Comparison between the control group and the cells groups (dead and viable) treated with the NE/PS5 and NE/PS10 complexes are shown in Fig. 6(c,d), respectively. Finally, Fig. 6e collects the data regarding all the studied cells groups. Important to stress that while the two clusters describing viable and dead cells show a slight overlap for the PDT treatment using the NE/PS5 complex the two clusters describing viable and dead cells treated with the NE/PS10 complex are quite separated (see Fig. 6e).

The PCA scatter plots of the 4T1 breast cancer cells showed in Fig. 6 demonstrate that the SERS spectra of all cells groups are reproductive even among spectra recorded from different groups or among spectra within the same cell group. This finding reinforces the need of a statistical approach as shown in Fig. 2, which incorporates robustness in the analysis of variation of integrated areas related to the vibrational modes of proteins and lipids.

## Discussion

Raman spectroscopy has been employed in identification of death mechanisms of various types of cells, such as leukemia cells^39^, T lymphocytes^40, 41^ and melanoma cells (MEL-28)^42^, among others^35, 43^. In all these reports physical effects, chemotherapeutic agents or other pharmaceutical substances were used to induce cell death events variants that showed different impacts onto cell molecular constitution. All those studies have found significant differences between Raman spectra of treated cells compared with untreated ones (control). In a recent report, Brauche *et al*.^43^ observed intensity increase in Raman peaks associated with DNA (at 795 and 1375 cm^−1^) in cells that underwent apoptosis and necrosis induced by exposing at different temperatures. However, the vibrational modes the authors found at 786 and 1315 cm^−1^, also assigned to DNA, showed opposite behavior, i.e. intensity reduction in both cases^43^. The authors^43^ also found that the Raman peak at 1375 cm^−1^ was practically absent in spectra recorded from control cells and enhanced in spectra of dead cells due to cellular nucleus condensation, which is associated with both cell death mechanisms. This conclusion was based on observations reported in other publications^40, 41^. Additionally, it was observed that the intensity of the 1003 cm^−1^ Raman peak, which is associated with phenylalanine, was increased in necrotic cells while being reduced in apoptotic cells. Moreover, the intensity of the 1437 cm^−1^ Raman peak, associated with lipids, was increased in apoptotic cells and reduced in necrotic ones. Notingher *et al*.^34^ and Verrier *et al*.^35^ also observed similar results while studying cell death via apoptosis. Both studies observed intensity reduction of Raman peaks associated with DNA (~788 cm^−1^) and proteins (1005, 1322, and 1342 cm^−1^) and intensity increase of Raman peaks associated with lipids (1301, 1449, and 1660 cm^−1^). Authors in refs 42 and 43 reported similar findings while investigating necrotic cells; reduction of Raman intensity peaks associated with lipids (935, 1085, and 1448 cm^−1^) and increase of Raman intensity peaks associated with proteins (642, 875, 1230, 1395, and 1425 cm^−1^). However, Raman peaks related to vibrational modes of DNA/RNA did not show similar behavior, i.e. intensity increase of Raman features peaking at 781, 985 and 1575 cm^−1^ and intensity reduction of Raman peaks at 667, 990 and 1305 cm^−1^. Despite the divergence of behavior observed for Raman bands of DNA/RNA it is possible to conclude that apoptosis is characterized by intensity decrease of Raman modes associated with proteins and intensity increase of Raman modes related to lipids. Moreover, necrosis leads to intensity increase of Raman modes associated with proteins and intensity decrease of bands related to lipids^34, 35, 43^.

Unfortunately, few reports discuss the possible origins of different trends related to Raman intensity variation observed in vibrational modes of lipids and proteins within cells. Verrier *et al*.^35^ argued that Raman intensity decrease associated with proteins, and observed during apoptosis, is due to reduction of protein content in apoptotic cells caused by activation of caspases. Worth mentioning that Brauche *et al*.^43^ proposed a similar explanation. In this line of argument, Raman intensity increase observed in the vibrational modes of proteins during necrosis is likely due to the release of proteins from necrotic nuclei into the cytosol. Also, according to those authors, the intensity increase observed in the 1375 cm^−1^ Raman signal may indicate changes in the architecture of chromatin triggered by HMGB1 release (chromatin binding protein that is relocated in necrotic cells). On the other hand, Kunapareddy *et al*.^42^ suggested that Raman intensity decrease associated with lipids would be consistent with coagulation necrosis. In necrosis, destruction of cytoplasmic structures and degradation of cell membranes occur in the first 24 hours and may be responsible for the observed reduced lipid content^42^. Thus, Raman intensity increase associated with proteins is directly correlated to the spectral analysis. In this regard all the recorded spectra were normalized, thereby decrease observed in one spectral component (e.g. lipids) leads to increase of another one (e.g. proteins). Despite this observation, the authors mentioned that there are spectral evidences indicating changes in protein conformation or composition.

Based on the above-presented background and taking into account the results shown in Fig. 2, revealing SERS intensity increase related to proteins and intensity decrease associated with lipids, it is possible to argue that necrosis is the main process responsible for cell death of the 4T1 breast cancer cells groups treated with PDT using the NE/PS5 and NE/PS10 complexes. This finding is in agreement with results reported in previous study^10^, where the efficacy of the PDT treatment of cancer cells (MCF7 and 4T1) and non-cancer cells (NIH/3T3 and MCF-10A) mediated by ClAlPc associated with polymeric nanoparticles (poly(methyl vinyl ether-co-maleic anhydride)) was investigated. Moreover, the authors reported that the employed PDT treatment induced cell death mainly by apoptosis when cells were irradiated with low energy density (0.48 J × cm^−2^) and necrosis when irradiated with high energy density (1.38 J × cm^−2^)^10^. For tumor cells following necrosis the loss of cell membrane integrity was confirmed by ethidium bromide staining^10^, a result in agreement with lipid reduction. Worth mentioning at this point that the energy density used in the present study was 10 J × cm^−2^, thus supporting the necrosis hypothesis as the dominant cell death mechanism in our case.

Though vibrational modes related to DNA/RNA do not exhibit an unique behavior while probing cell death by apoptosis or necrosis intensity reduction of vibrational modes associated with phosphodiester bonds (O-P-O) of DNA structure was recently assigned to cellular apoptosis^38^. In this report, the authors used Gold nanoparticles to enhance Rayleigh and Raman signal in human oral cancer cells (SH-3) to investigate cellular apoptosis induced by hydrogen peroxide treatment. During apoptosis the intensity of the vibrational mode peaking at 838 cm^−1^ (O-P-O) decreased while the intensity of the Raman feature peaking at 1585 cm^−1^ (N_7_-H in guanine/adenine bases) increased. According to the authors this trend is associated with fragmentation of DNA structure in the phosphodiester bonds, thus exposing DNA bases to the Gold nanoparticles plasmonic field. Our findings differ from those reported in ref. 38. as we found the 813 cm^−1^ peak intensity increasing while reducing the 1565 cm^−1^ band intensity, suggesting that DNA fragmentation mainly occurs by breakage of base pairs rather than due to the breaking of phosphodiester bond. Separation of base pairs exposes the backbone of the DNA dorsal to the Silver substrate, resulting in enhancement of the Raman signal (SERS effect). The highest intensification of the SERS signal for the 813 cm^−1^ band was observed in the dead cells group treated with the NE/PS5 complex, which is assumed to be due to the highest degree of DNA fragmentation in this cellular group. In this case, in addition to separation of the DNA base pairs, breakage of the phosphodiester bonds may also occurred. Moreover, besides the spectral changes associated with DNA fragmentation, in the SERS spectra of the dead cells groups it was also observed a intensity decrease of the Raman peaks at 520 and 549 cm^−1^, associated with vibrations of disulfide bonds (-S-S-) of proteins. This behavior can be explained considering the breakdown of intramolecular disulfide bonds responsible for proteins tertiary/quaternary folding^38^.

It is well known from the literature that the key biological catalyst of disulfide bonds is ERO-α (endoplasmic reticulum oxidoreductase alpha) enzyme, which is produced in endoplasmic reticulum^44^. A recent study conducted with breast cancer patients reported a significant increase of the ERO-α enzyme activity in 4T1 breast cancer cells while compared to non-cancerous cells/tissues^45^. Therefore, it is expected that cancer cells show high concentration of biomolecules presenting disulfide bonds. Additionally, it was noticed a drastic reduction of tumor growth in 4T1 breast cancer cells when they are deprived of the ERO-α^46^. The authors concluded that the ERO-α enzyme contributes to the suppression of anti-tumor immunity by regulating G-CSF cytokines and CXCL1/2 via facilitating proteins folding, suggesting that inhibition of the ERO-α enzyme may difficult cancer progression.

Figure 4(a,b) show the ratio *A*_*viable*_/*A*_*dead*_ collected for different vibrational modes associated to proteins and lipids while using the NE/PS5 and NE/PS10 complexes, respectively. Worth mentioning that even under 10 J/cm^2^ illumination, either using the NE/PS5 complex or the NE/PS10 complex, cells undergo necrosis as previously reported^9^. Moreover, the higher the PS doses the lower the viable cell population after performing the PDT. Therefore, vibrational modes peaking at 608, 846, 1003 and 1500 cm^−1^ for proteins and 718 and 1231 cm^−1^ for lipids are reasonable candidates for monitoring cell population ratio as shown in Fig. 4(a,b), respectively. Among the selected vibrational modes for monitoring viable and dead cells the highest discrimination is 608 cm^−1^ for proteins and 1231 cm^−1^ for lipids, as shown in Fig. 4(a,b), respectively. Additionally, note in Fig. 4(a,b) the pair of vibrational modes peaking at 608 cm^−1^ (proteins) and 1231 cm^−1^ (lipids) seems to be a good candidate for monitoring viable/dead cell population, given the values of *A*_*viable*_/*A*_*dead*_ below 2 (NE/PS5) and 1 (NE/PS10) and above 2 (NE/PS5) and 1 (NE/PS10) for proteins and lipids, respectively. This observation fully agrees with increasing SERS line intensity for proteins and decreasing SERS line intensity for lipids contents in cells undergoing necrosis. Therefore, we claim that the corresponding pair of vibrational modes (for proteins and lipids) are potentially the best candidates for quantitative follow up of cell survival after the PDT treatment employed in the present study. This finding is a key point while using spatial infrared (IR) thermography imaging in oncology^47, 48^. In the context of thermographic IR imaging quantitative capability represents the forefront trend of research in this area, particularly because of the non-invasive characteristic of the technique and advances in computing and image-analysis software in the last decades^47, 48^. However, identification of IR bands that can be used to enhance the IR imaging performance is challenging, and the present report offers a robust approach to fulfill this requirement. In this regard, a previous screening of promising vibrational modes that can offer superior discrimination of neoplastic cells can be performed using the approach introduced in the present study. In principle, the approach herein proposed can be extended to a wide variety of neoplastic cells and tissues, thus fostering the development and transferring the spatial infrared thermography imaging technology from the bench to the clinic.

Though extremely useful for future cancer diagnosis development and follow up protocols the explanation of the observed SERS spectral variations among control cells, viable cells and dead cells groups is far from to be a simple task. However, using a robust statistical tool, we can infer that significant intensity increase in the Raman vibrational modes associated with proteins in viable cells may be related to activation of intracellular defense mechanisms. Among these mechanisms it has been highlighted alteration on protein synthesis encoded by genes stimulated or suppressed during cellular stress caused by the PDT treatment^3^. In this process, genes encoding inducing signals of cell proliferation promotes production of proteins (growth factors, growth factor receptors, transcription factors, and apoptosis regulators) responsible for cell proliferation. When cell proliferation is stimulated several molecular mechanisms are activated in an attempt to control the process of cell division. New proteins are synthesized to account for those mechanisms, such as the cyclin proteins. In dead cells, the production of proteins is suppressed and, therefore, dead cells show less protein content while compared to viable cell. Thus, the significant intensity increase we found in the Raman bands assigned to proteins in the viable cells, as observed in the recorded SERS spectra, are likely related to activation of defense mechanisms induced by oxidative stress due to the employed PDT treatment. A careful analysis of the SERS spectra shown in Fig. 2 reveals that it is possible to observe spectral variations among the three cells groups studied here (control, dead, and viable) and between the two cells groups treated with different PS doses (NE/PS5 and NE/PS10 complexes). Furthermore, the employed PCA tool adds robustness to our conclusions. Figure 6(a,e) show that the data points collected from the dead cells fall in well separated clusters, according to the PS dosage received (treatment with the NE/PS5 or NE/PS10 complex). Moreover, the data points from the dead cells group treated with the NE/PS10 complex (solid triangles) are located closer to the control group than the dead cells group treated with the NE/PS5 complex (open triangles). This finding may indicate that the dead cells group treated with the NE/PS10 complex undergoes less biochemical changes before the death mechanism is triggered. On the other hand, in the viable cells groups it is observed a superposition of data points on the PCs space, as shown in Fig. 6(b,e). However, compared with the viable cells group treated with the NE/PS5 complex we found that the data points related to the viable cells group treated with the NE/PS10 complex is farther from the data points related to the control group. As far as the employed PDT protocol is concerned this finding may indicate that the viable cells group presents higher biochemical change when treated with the highest PS doses (NE/PS10 complex).

## Conclusion

Surface Enhanced Raman Spectroscopy (SERS) was successfully used to investigate breast cancer cells treated with photodynamic therapy (PDT). The photosensitizer (PS) used in the PDT treatment was formulated as nanoemulsions (NE/PS) with two different PS (chloroaluminumphthalocyanine) contents (5 and 10 µmol × L^−1^). Analysis of the SERS data using Principal Component Analysis (PCA) showed that the employed PDT treatment with different PS concentrations leads to significant biochemical changes related to proteins, lipids, and DNA. Changes in SERS intensity related to proteins and lipids indicated necrosis as the main cellular death mechanism after the PDT treatment. Also, compared to untreated cells (control group), the SERS spectra collected from dead cells groups showed significant changes in the vibrational modes of DNA. The increased intensity of vibrational modes of phosphodiester bond (O-P-O) plus the intensity reduction of the SERS bands related to the guanine/adenine bases suggested that after the PDT treatment DNA fragmentation mainly occurs by breakage of its base pairs. Additionally, while compared with control group, the SERS data recorded from viable cells groups showed that the significant intensity increase of the vibrational modes related to proteins are likely associated with cellular defense mechanisms induced by oxidative stress due to the PDT treatment. Moreover, the PCA analysis indicated that biochemical changes observed in dead cells treated with the NE/PS10 complex was lower than changes observed in dead cells treated with the NE/PS5 complex. This finding can be understood as long as the death cell mechanism (necrosis) in cells treated with the former (NE/PS10 complex) is triggered at earlier times compared with the treatment performed with the latter (NE/PS5 complex). Finally, we found the SERS modes 608 and 1231 cm^−1^, ascribed respectively to proteins and lipids, are the best candidates for follow up of 4T1 cells survival subjected to PDT treatment. We claim that this finding is a key contribution to the development of quantitative infrared thermography imaging.

## Methods

### Nanoemulsion of ClAlPc

Nanoemulsions containing the photosensitizer (PS: ClAlPc) were prepared from solubilization of ClAlPc in ethanol 99 °GL at 1.7 mmol × L^−1^. Then, 12 g of a mixture comprising 75% of Cremophor ELP® and 25% of castor oil was added to the previous ClAlPc solution, producing a mixture containing 40 µmol × L^−1^ of ClAlPc. Next, the ethanol was removed at 100 °C under gentle magnetic stirring (300 rpm for 15 minutes). After cooling the mixture down to room temperature 70 mL of culture medium was added under mild agitation for extra 15 minutes. Then, culture medium was added to fix the final ClAlPc concentration at 5 and 10 µmol × L^−1^ of AlClPc and the final complexes were labeled NE/PS5 and NE/PS10, respectively.

### Culture and treatment of breast cancer cells

Mouse mammary glands cancer cells (line 4T1 ATCC® CRL-2539) were grown in Dulbecco’s modified Eagle medium (DMEM) supplemented with 10% fetal bovine serum and 1% antibiotic solution (100 IU mg × L^−1^ penicillin and 100 mg × mL^−1^ streptomycin). The cells were maintained at 37 °C in a humidified atmosphere of 5% CO_2_ for 24 hours. Then, the cells were divided into two main groups: control group (kept under the same growing condition for another 24 hours) and treated groups (submitted to the PDT treatment using NE/PS5 or NE/PS10 complex). Afterwards, all cells groups were frozen in liquid Nitrogen.

For the PDT treatment, cells were separated into two groups and treated with the NE/PS5 or NE/PS10 complex. In both cases, cells were exposed to the complexes for 15 minutes in dark and washed twice with phosphate-buffered saline (PBS). Next, cells were irradiated with LED lamp (660 nm) at an energy density of 10 J × cm^−2^ for 10 minutes. After treatment, cells were cultured for 24 hours under the culture condition described above. After 24 hours supernatant cells (dead cells) were separated from surviving cells (viable cells) and both groups were frozen in liquid Nitrogen.

### SERS substrate and sample preparation for SERS measurement

Electrodeposited films of Silver were used as SERS substrate. The Silver films were prepared by electrodeposition of Silver ions onto Silver electrodes. After cleaning and polishing the Silver electrodes were immersed within a solution of 200 mL distilled water containing 22 mg of AgNO_3_. Next, the as-produced electrodes were subjected to a potential difference of 3 V, electric current of approximately 1000 µA for a period of 1 hour. Then, the negative electrodes (SERS substrates) were immediately dried under Nitrogen flow.

The frozen cells were ground and stirred to obtain a liquid and homogeneous solution. Then, 10 µL of the cells were deposited onto the Silver film previously prepared. Next, the films were dried under Nitrogen flow and kept within sealed containers with plastic film until they were used for measurements. Raman measurements were performed shortly after the probing samples were prepared (up to 4 hours after preparation) to avoid film’s oxidation and cells’ degradation.

### Instrumentation

For acquisition of Raman spectra a triple spectrometer Jobin Yvon model T64000 equipped with a 1800 groves/mm grating and a CCD (Charge-Coupled Device) 2048 × 512 pixels cooled Nitrogen was used. The SERS measurements were performed with the macro-Raman system, in which the laser beam was focused onto the sample using a cylindrical lens with an excitation power of 0.1 W × cm^−2^, to prevent sample heating and damaging cells. The 488 nm Argon laser line was used as the excitation source.

### Data analysis

Analysis of the Raman data was performed with the LabSpec 4.10 software. The background signal was subtracted from the recorded Raman spectra and the resulting data were individually normalized with respect to the integrated area in the range of 400–1800 cm^−1^. To improve analysis of spectral differences among the five cells groups SERS measurements were carried out on 30 samples for each group. Next, Principal Component Analysis (PCA) was employed in the analysis of all 150 spectra (30 spectra for each 5 cells groups). The PCA was implemented within the MINITAB® software^49^.

### Data Availability

All data generated or analysed during this study are included in this published article.
